# Delgocitinib 20 mg/g cream for the treatment of lichen planopilaris: A case report

**DOI:** 10.1016/j.jdcr.2026.07.026

**Published:** 2026-07-23

**Authors:** Mina Farah, Nikkia Zarabian, Maher Ali, Joseph Zahn, Adam Friedman

**Affiliations:** a Department of Dermatology, The George Washington University School of Medicine and Health Sciences, Washington DC; b Department of Pathology, The George Washington University School of Medicine and Health Sciences, Washington DC

**Keywords:** delgocitinib cream, hair loss, JAK inhibition, lichen planopilaris, off-label, scarring alopecia

## Case report

Lichen planopilaris (LPP) is a primary lymphocytic cicatricial alopecia in which an inflammatory infiltrate targets follicular antigens in the hair follicle bulge, resulting in CD8^+^ T cell-mediated follicle destruction and replacement with fibrous tissue.^1^ This process is driven by T helper (Th)1-mediated upregulation of IFN-γ signaling, interleukin 17, interleukin 21, and interferon-inducible chemokines and results in permanent scarring hair loss.^1^^,^^2^ Clinically, LPP may present with perifollicular hyperkeratosis, erythema, and scaling, which over time may lead to patchy hair loss.^1^

There are currently no on-label therapies for LPP, with intralesional and topical corticosteroids as the most commonly prescribed therapies, followed by oral doxycycline (200 mg daily) and hydroxychloroquine (200 mg once or twice daily).^1^ Additionally, off-label systemic Janus kinase (JAK) inhibitors (JAKis) have shown some efficacy in treating scarring alopecia, including LPP.^3^ Tofacitinib, a JAK1/3 inhibitor, has been cited as the most effective JAKi in treating scarring alopecia, at doses ranging from 10 to 15 mg/day.^1^^,^^3^ In addition to systemics, topical JAKis, such as tofacitinib and ruxolitinib creams, a JAK1/2 inhibitor, have shown promise in treating scarring alopecia, including frontal fibrosing alopecia (FFA) and discoid lupus erythematosus.^4^, ^5^, ^6^ Delgocitinib cream, a newer topical pan-JAKi, has also been reported to treat scarring alopecia but has not been evaluated for LPP and may offer a promising therapeutic approach through its unusual mechanism of action.^4^^,^^5^ We therefore present a case of twice-daily topical delgocitinib 20 mg/g cream as a novel treatment modality for LPP.

### Case 1

**Fig. 1:**
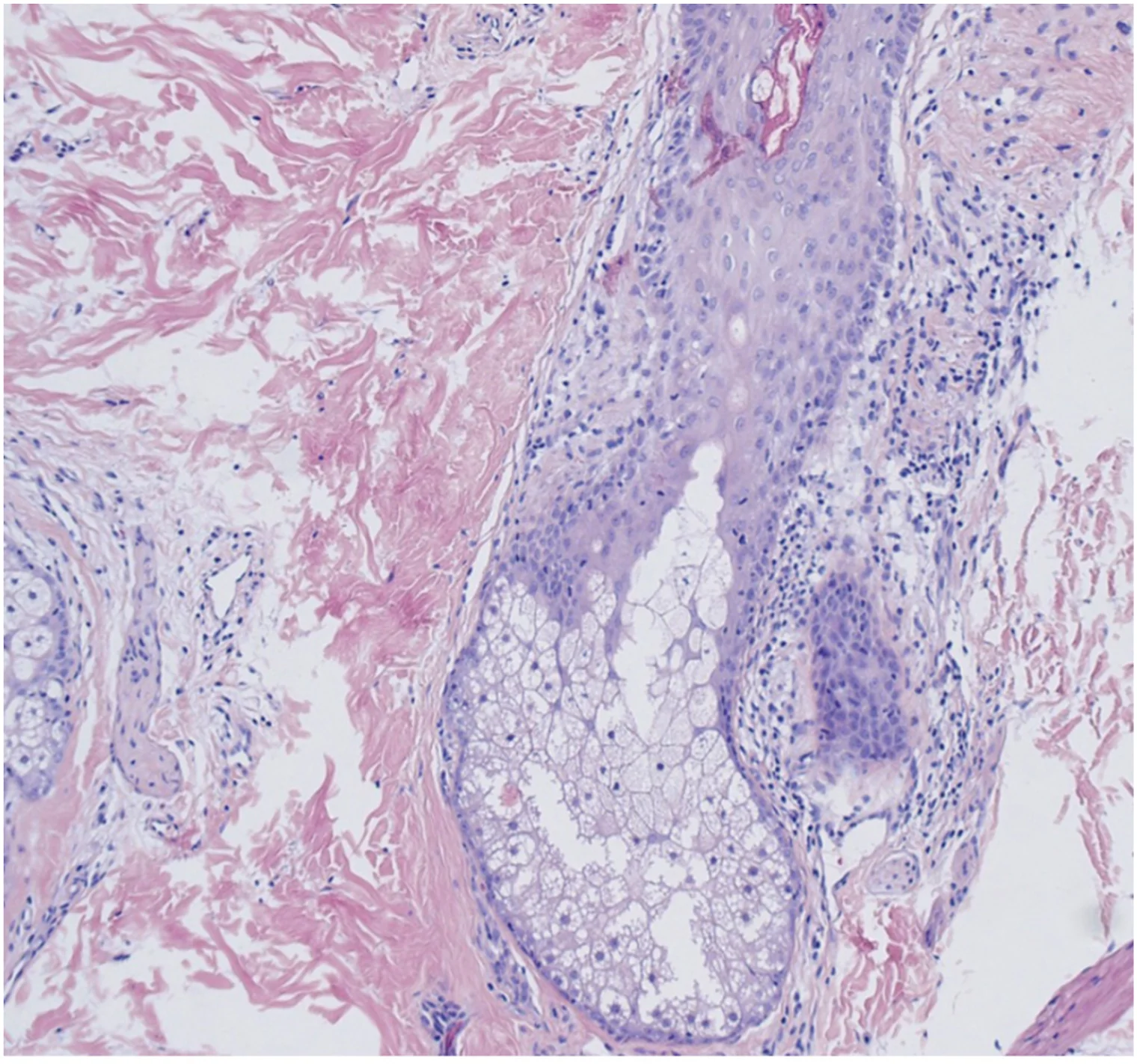
Histology of lichen planopilaris scalp biopsy. (Original magnification: ×10).

A 20-year-old healthy woman presented with a pruritic, ill-defined pink patch on the posterior scalp, characterized by central and perifollicular scaling with associated hair loss and broken hairs of varying lengths. Given the broad differential diagnosis, including alopecia areata, LPP, discoid lupus, and trichotillomania, a punch biopsy was obtained. Histopathologic examination revealed a mild spongiotic-to-early lichenoid infiltrate around the level of the isthmus of the hair shafts, without evidence of mucin, interface dermatitis, or fungal elements, consistent with LPP (Fig. 1).

**Fig. 2:**
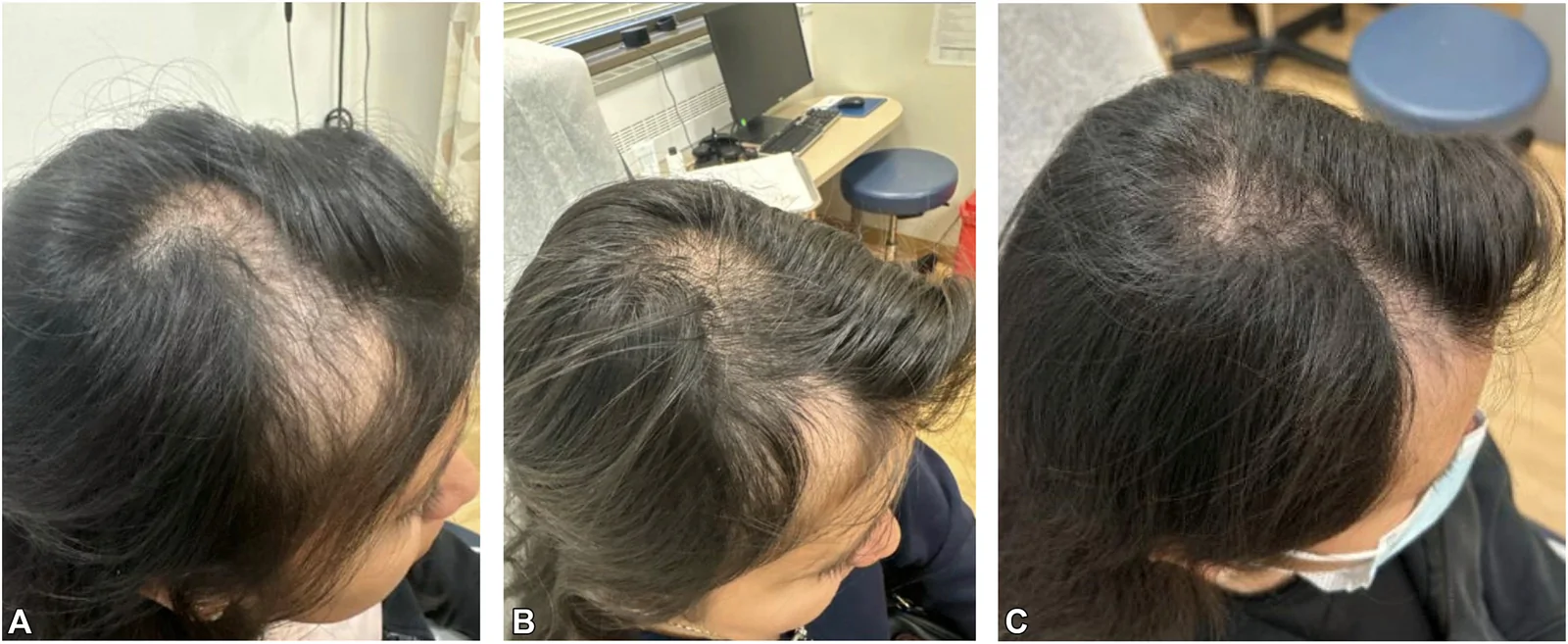
Clinical presentation of lichen planopilaris **(A)** prior to initiation of delgocitinib treatment, **(B)** 2 months, and **(C)** 4 months posttreatment initiation.

Initial management included intralesional triamcinolone acetonide 5 mg/mL, topical clobetasol 0.05% scalp solution, and intermittent ketoconazole 2% shampoo. Due to experiencing minimal improvement and preferring nonsystemic options, the patient subsequently discontinued previous treatments and initiated off-label topical delgocitinib 20 mg/g cream twice daily to the affected area. By 2 months, she demonstrated marked improvement in hair density, with near-complete regrowth observed at 4 months of treatment with twice-daily delgocitinib 20 mg/g cream (Fig. 2).

## Discussion

This case represents the first reported successful treatment of LPP with topical delgocitinib cream and highlights a promising topical therapeutic option. Given the typically limited potential for hair regrowth in LPP, this report introduces a novel modality that may help broaden the treatment landscape for localized disease and provide further insight into the inflammatory mediators involved in LPP.

Currently approved for the treatment of moderate-to-severe, chronic hand eczema, delgocitinib is a topical pan-JAKi that targets JAK1, JAK2, JAK3, and tyrosine kinase 2.^5^^,^^7^ It thereby modulates multiple cytokine pathways, including Th1, Th2, Th17, and Th22 inflammatory cascades.^5^^,^^7^ Given the role of JAK-dependent cytokines and interferon signaling in the pathogenesis of LPP, the degree and breadth of JAKi achieved with delgocitinib cream provide a plausible therapeutic mechanism.^2^ By attenuating these inflammatory pathways, delgocitinib may offer a treatment modality targeted to the inflammatory profile of LPP, thereby reducing follicular destruction and facilitating hair regrowth.

Within the realm of scarring alopecia, topical delgocitinib has been reported to treat FFA. A recent randomized, double-blind, phase 2a trial of delgocitinib 20 mg/g cream versus vehicle cream in 30 adult female patients with FFA demonstrated marked disease improvement by 12 weeks, with significant downregulation of C–X–C motif chemokine ligand 9, a Th1-related biomarker implicated in the pathogenesis of FFA, and a significant mean improvement in the transcriptomic profile.^4^ In contrast, affected areas treated with vehicle cream worsened.^4^ Beyond FFA, delgocitinib cream is currently being investigated for the treatment of discoid lupus erythematosus, as the JAK-STAT signaling pathways play a crucial role in discoid lupus erythematosus pathophysiology, further highlighting the applicability of delgocitinib in the management of scarring alopecia.^5^

Other topical JAKis have also demonstrated efficacy in LPP. Chen et al^8^ reported tofacitinib cream, applied once to twice daily or several times weekly, as an alternative to topical corticosteroids, with clinical improvement observed after a mean of 6 months.^8^ Similarly, Williams et al^6^ described ruxolitinib 1.5% cream, used twice daily or every other day, as an adjunctive therapy associated with a 25% to 75% reduction in LPP activity index scores. While effective and well-tolerated, both topical ruxolitinib and tofacitinib carry Food and Drug Administration black box warnings for malignancy, serious infections, mortality, major adverse cardiovascular events, and thrombosis.^1^ However, a robust meta-analysis including over 20,000 patients demonstrated no increased risks of mortality, major adverse cardiovascular events, or venous thromboembolism with JAKis.^9^ Despite reassuring data, perceived safety concerns persist and have affected prescribing patterns.^9^^,^^10^ In contrast, topical delgocitinib cream lacks this warning and consequently may appeal to a broader population. Additionally, through pan-JAK inhibition, delgocitinib cream targets a broader range of inflammatory mediators than other topical JAK inhibitors, which may enhance treatment response.^5^ Although direct comparisons of delgocitinib cream to other topical JAKis are lacking, an in vivo murine study demonstrated more potent inhibition of the inflammatory response with oral delgocitinib compared with oral tofacitinib, supporting its potentially greater anti-inflammatory effect.^5^

While topical JAKis have been previously explored in LPP, this case is the first to report the efficacy and tolerability of topical delgocitinib, highlighting the utility of pan-JAK inhibition in LPP management. Although limited to a single report, these findings support further investigation, including head-to-head comparisons of topical JAKis.

## Conflicts of interest

Author Farah work is funded through independent research grants from Incyte and Johnson & Johnson. Author Zarabian work is funded through an independent research grant from Galderma. Dr Friedman is a speaker/consultant for Regeneron, Novartis, Sanofi, Pfizer, LEO Pharma, and Lilly. Dr Zahn holds Regeneron stock and stock options and is an employee of Regeneron. Dr Ali has no conflicts of interest to declare.
